# Supplementary material for: Ultralong π-Conjugated Bis(terpyridine)metal Polymer Wires Covalently Bound to a Carbon Electrode: Fast Redox Conduction and Redox Diode Characteristics
Source: Molecules. 2021 Jul 14;26(14):4267. doi: 10.3390/molecules26144267 (PMC8307305; doi:10.3390/molecules26144267)
Supplement: Supplementary file 1 [file molecules-26-04267-s001.zip › molecules-1281712-supplementary.pdf]

Supplementary Materials

Ultra-long π-Conjugated Bis(terpyridine)metal Polymer Wires Covalently Bound to a Carbon Electrode: Fast Redox Conduction and Redox Diode Characteristics

Kuo-Hui Wu[1,2,*], Ryota Sakamoto[1,3], Hiroaki Maeda[1,4], Eunice Jia Han Phua[1], Hiroshi Nishihara[1,4*]

[1] Department of Chemistry, Graduate School of Science, The University of Tokyo, 7-3-1, Hongo, Bunkyo-ku, Tokyo 113-0033, Japan
[2] Department of Chemistry, National Central University, 300 Jung-Da Rd. Jhong-Li 32001, Taiwan
[3] Department of Energy and Hydrocarbon Chemistry, Graduate School of Engineering, Kyoto University, Nishikyo-ku, Kyoto 615-8510, Japan
[4] Research Center for Science and Technology, Tokyo University of Science, Chiba 278-8510, Japan Tokyo University of Science
* Correspondence: wukuohui@cc.ncu.edu.tw (K.-H. W); nisihara@rs.tus.ac.jp (H.N.)

	page
EXPERIMENTAL SECTION	S2
Electropolymerization of 1-Fe by consecutive cyclic scans	S4
Raman spectrum of Fe polymer wire, **2-Fe**, and its peak assignment	S5
Cyclic voltammograms of **2-Fe** and **2-Ru** at various scan rates	S6
DFT calculation	S7
Evaluation of the length of **2-Fe** and **2-Ru** blocks in **3**	S9
Evaluation of the length of **2-Fe** and **2-Ru** blocks in **4**	S10

EXPERIMENTAL SECTION

Materials. Iron(II) tetrafluoroborate hexahydrate ($Fe(BF)_4 \cdot 6H_2O$, 97%, Aldrich), 2,2':6',2"-terpyridine (>98%, TCI), ruthenium(III) chloride hydrate (38.9% Ru basis, Furuya Metal), perchloric acid ($HClO_4$, 60%, Wako Pure), acetonitrile (MeCN, >99.9%, Kanto Chemical), ethanol (95%, Kanto Chemical). Tertrabutylammonium perchloride (Bu_4NClO_4, >98%, TCI) was recrystallized from ethanol. 4'-(4-Anilino)-2,2':6',2"-terpyridine (NH_2-Ph-tpy), bis(4'-(4-anilino)-2,2':6',2"-terpyridine)iron(II) tetrafluoroborate ($Fe(NH_2\text{-Ph-tpy})_2(BF_4)_2$, **1-Fe**) and bis(4'-(4-anilino)-2,2':6',2"-terpyridine)ruthenium(II) hexafluorophosphate ($Ru(NH_2\text{-Ph-tpy})_2(PF_6)_2$, **1-Ru**) were synthesized according to literature procedures.[S1] (*E*)-1,2-bis(4-([2,2':6',2"-terpyridin]-4'-yl)phenyl)diazene (tpy-AB-tpy, **5**) was synthesized following a literature procedure.[S2] Random length ((*E*)-1,2-bis(4-([2,2':6',2"-terpyridin]-4'-yl)phenyl)diazene)iron(II) tetrafluoroborate polymer wire ($[(\text{tpy-AB-tpy})Fe]_n$, **6**) was synthesized by reacting $Fe(BF)_4 \cdot 6H_2O$ with **5** in MeCN and used without further treatment.

Instruments. Electrochemical measurements were carried out in a three-electrode cell equipped with a glassy carbon disk (3 mmϕ) working electrode, a Pt coil counter electrode and a Ag/AgCl (in aqueous solution) or a Ag/Ag^+ (in nonaqueous solution) reference electrode with ALS 750A and 650DT electrochemical analyzers (BAS Co., Ltd.). Raman spectra of **2-Fe** on GC and the chemically synthesized **5** on a silicon wafer were obtained using an NRS-1000N laser Raman spectrophotometer (JASCO). The cross- section image of **2-Fe** and thickness of polymer wire films were measured using VHX-100 optical microscope with NH-Z75 lens (KEYENCE). Topographic and phase images of atomic force microscopy (AFM) were obtained on an Agilent Technologies 5500 Scanning Probe Microscope.

Electrosynthesis of polymer wires 2, 3, 4. Electrosynthesis was carried out in a three-electrode electrochemical cell using a piece of glassy carbon (GC) with the diameter of 3 mm sealed in a glass tube (for electrochemical study) or a piece of GC disk with the diameter of 10 mm (for Raman spectroscopy and AFM) as the working electrode, a platinum coil as the counter electrode, a Ag/AgCl electrode as the reference electrode and a 1 mM water-acetonitrile-$HClO_4$ solution of $M(NH_2\text{-Ph-tpy})_2$ (**1-M**) as the reaction solution. This solution was prepared by dissolving 0.01 mmol of **1-M** in 5 mL of acetonitrile, followed by adding 5 mL of ultra-pure water and 0.109 mL of 60% $HClO_4$ solution. For synthesizing the polymer wires **2-Fe** and **2-Ru**, consecutive potential scans are carried out between -0.5 V and +1.4 V for **1-Fe** and between -0.3 V and +1.6 V for **1-Ru**, respectively, at a scan rate of 0.1 V s^{-1}. The resulting modified electrodes were washed by ultra-pure water, acetonitrile and acetone successively, and immersed in acetonitrile for overnight.

Electrochemical dissociation of $Fe(tpy)_2$ polymer wires from 2-Fe and synthesis of $Fe(tpy)_2$ monolayer. The glassy carbon electrode modified with $Fe(tpy)_2$ polymer wires, **2-Fe**, was set in a three-electrode cell as the working electrode along with an Ag/AgCl reference electrode and a Pt counter electrode. A 0.1 M MeOH-KOH solution was used as the electrolyte solution. The dissociation was carried out by applying oxidation potentials on the working electrode sweeping from 0.6 to 1.4 V vs. Ag/AgCl at a scan rate of 0.1 V s^{-1} for 80 cycles. After this electrochemical treatment, the electrode was washed with MeOH, water, acetone and $CHCl_3$ with ultasonication for 10 min sequentially.

The electrochemically dissociated modified electrode was immersed in a 0.5 M ethanol-$Fe(BF_4)_2 \cdot 6(H_2O)$ solution for 1 hour. The electrode was then washed by ethanol, water and ethanol sequentially and dried by nitrogen blowing. This iron-solution-treated electrode was immersed in a 0.1 M 2,2':6',2"-terpyridine solution in chloroform for 12 hours and then washed with chloroform for three times and dried by nitrogen blowing.

DFT calculation. The DFT calculation is performed at the B3LYP level by Gaussian 09w. The basis set of Fe is LANL2DZ and that of C, N, H is 6-31g(d).

References

(S1) Storrier, G. D.; Colbran, S. B.; Craig, D. C. *J. Chem. Soc. Dalton Trans.*, **1997**, 3011.

(S2) Yutaka, T.; Kurihara, M.; Nishihara, H. *Mol. Cryst. Liq. Crys. A*, **2000**, *343*, 193.

Electropolymerization of 1-Fe by consecutive cyclic scans

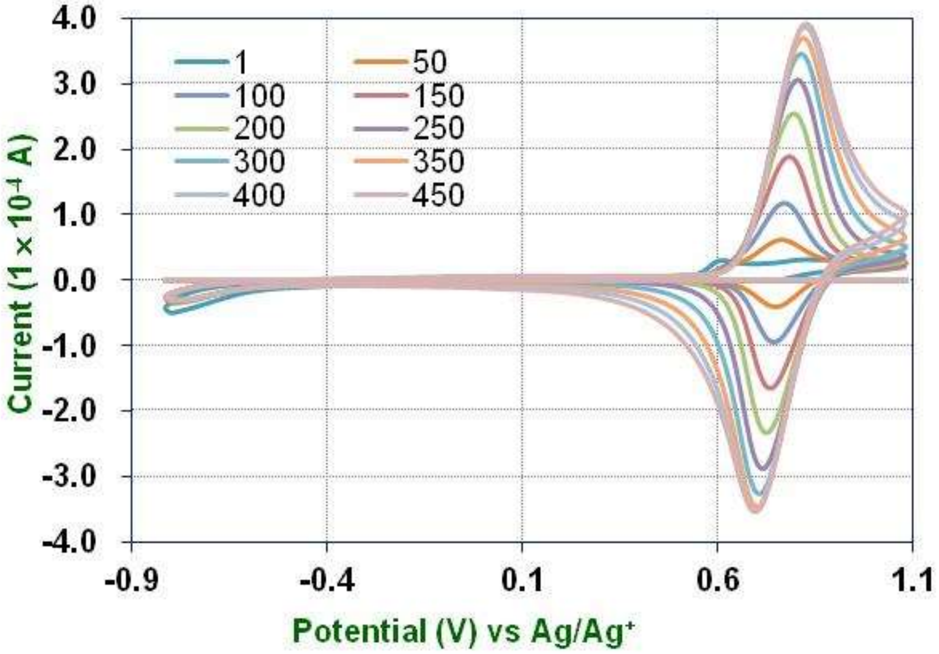

Figure S1. Cyclic voltammetry of **1-Fe** (1 mM) at a GC electrode in MeCN-H_2O-$HClO_4$ at a scan rate of 0.1 V s^{-1}. Numbers in the figure refer to those of the cyclic scans.

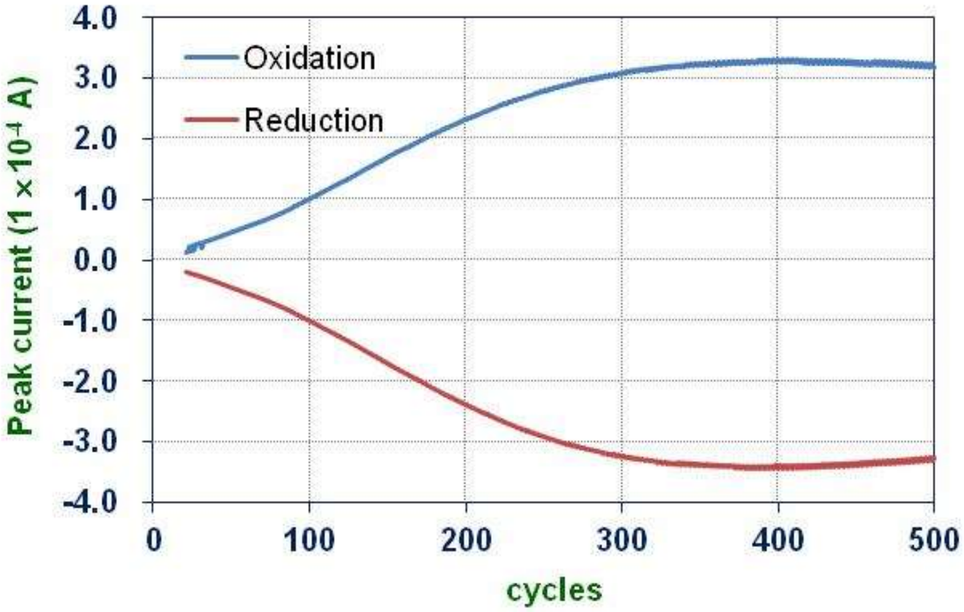

Figure S2. Relation of the oxidation and reduction peak currents in the cyclic voltammograms for electropolymerization of **1-Fe** (1 mM) at a GC electrode in MeCN-H_2O-$HClO_4$ at a scan rate of 0.1 V s^{-1} versus the number of the cyclic scans.

Raman spectrum of 2-Fe and its peak assignment

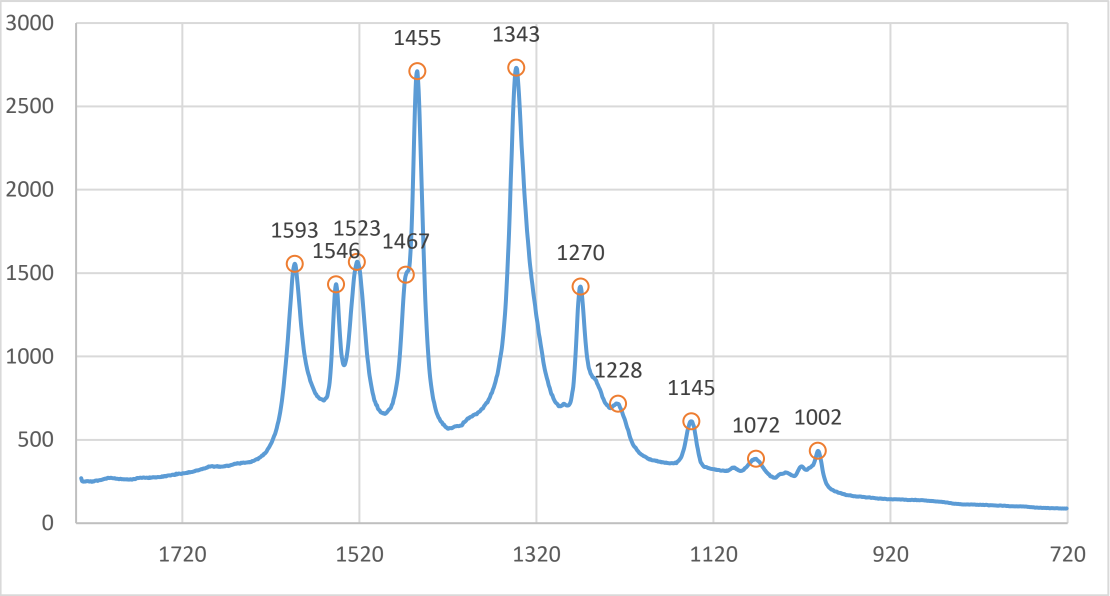

Figure S3. The Raman spectrum of **2-Fe** on glassy carbon.

Assignment of the peaks

1593	C=N (pyridine)
1546	C=C (pyridine)
1523	C=C (phenyl ring)
1467	C=C (phenyl ring)
1455	N=N (diazo)
1343	C-C (Ar-Ar)
1270	C-C (pyridine-pyridine)
1145	Ar-N (diazo)
1002	Fe-py

Cyclic voltammograms of 2-Fe and 2-Ru at various scan rates

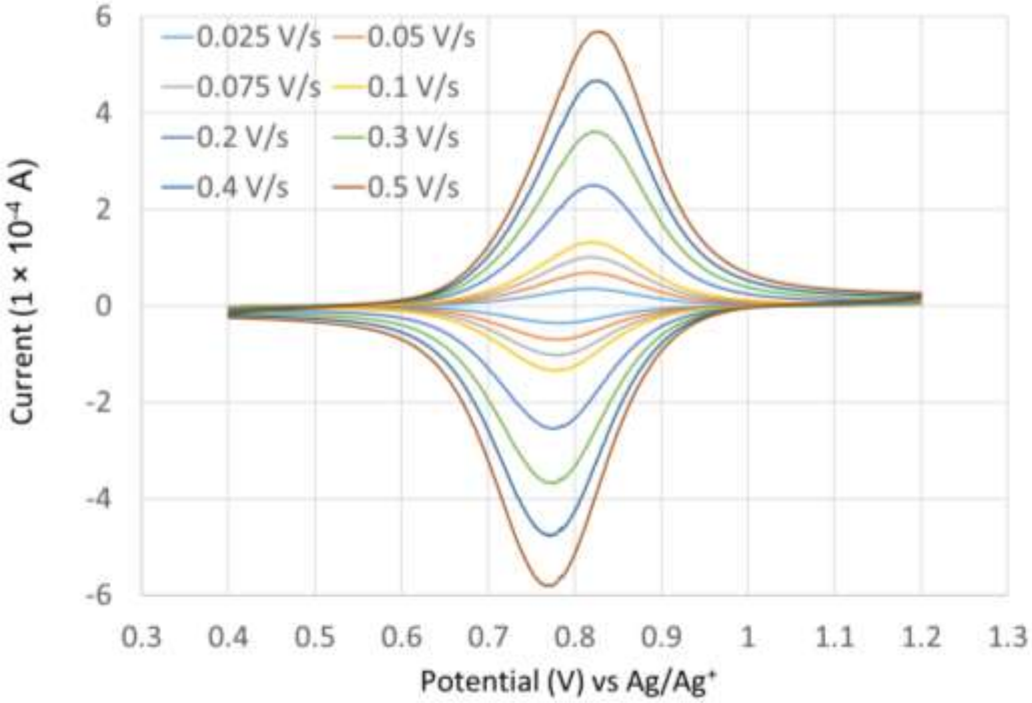

Figure S4. Cyclic voltammograms of **2-Fe** on GC in 1.0 M Bu_4NClO_4-MeCN.

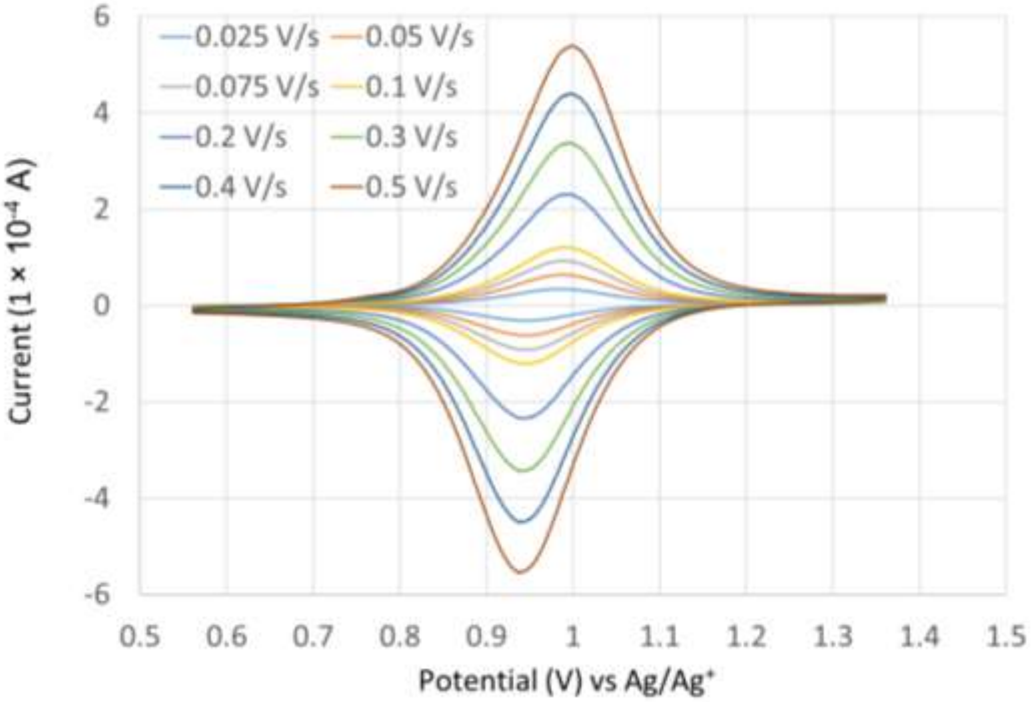

Figure S5. Cyclic voltammograms of **2-Ru** in 1.0 M Bu_4NClO_4-MeCN.

DFT calculation

From the optimized modelling structure (Figures S4, S5) in which a monolayer wire anchored on carbon, the distance between the two carbon atoms in the three-membered ring anchor is 2.204 Å, which is much longer than a covalent C–C single bond, and the nitrogen atom is drawn close to the graphite plane due to the strong strain of the small ring and the distorted graphite (Figure S5). Given that several atomic orbitals in the ring are not strongly restricted in σ bonds and are made available to be mixed into the π-orbitals of the wire and the orbitals of the electrode, the distorted ring contributes to the π-orbital of the wire, extending it to the electrode (HOMO- 2) (Figure S6). This orbital comprises the d_{xz} orbital of the iron, the π-orbitals of the pyridine and the phenyl ring, the p_x orbital of the nitrogen, and the orbital of graphite near the anchor. This π-orbital expansion between the complex and the electrode greatly reduces the energy barrier of electrons transporting between them, resulting in the high redox conductivity of this polymer wire. The HOMO and HOMO-1 are orbitals solely belonging to the graphite (Figure S6).

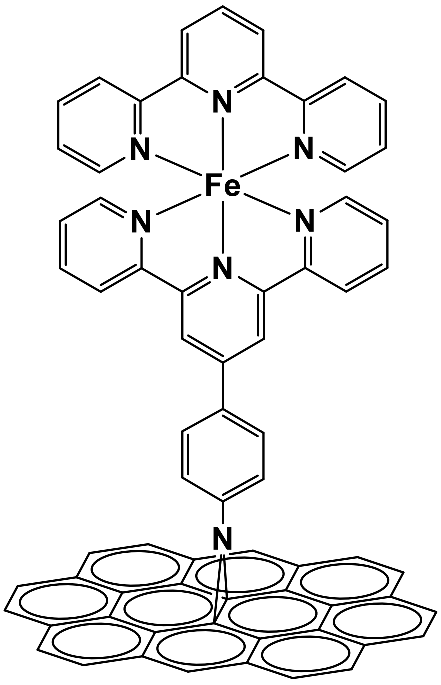

Figure S6. The chemical structure for the DFT calculation.

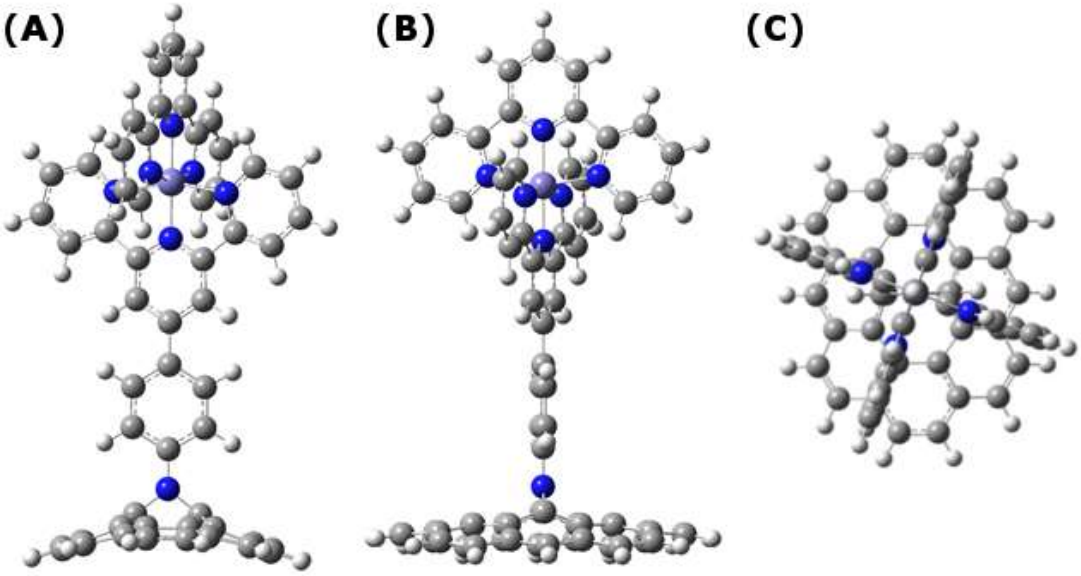

Figure S7. (A) and (B) Side view images of the optimized structure. (C) Top view image of the structure.

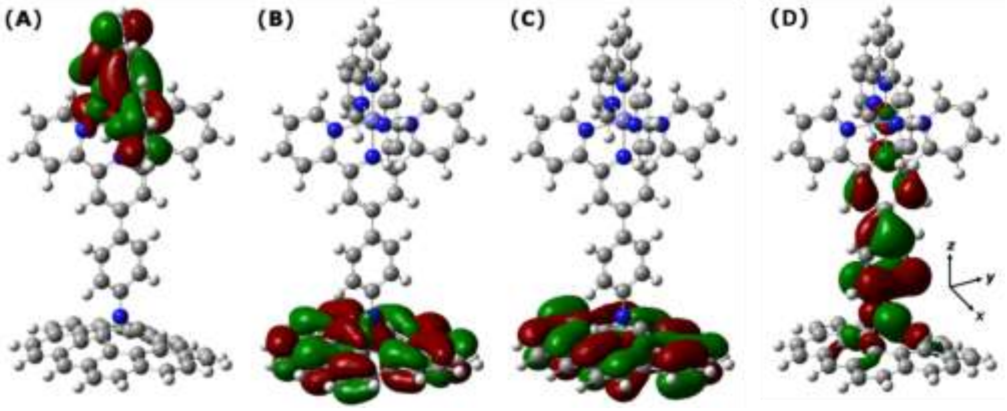

Figure S8. The LUMO (A), the HOMO (B) and the HOMO–1 (C) of the optimized structure.

Evaluation of the length of 2-Fe and 2-Ru blocks in 3

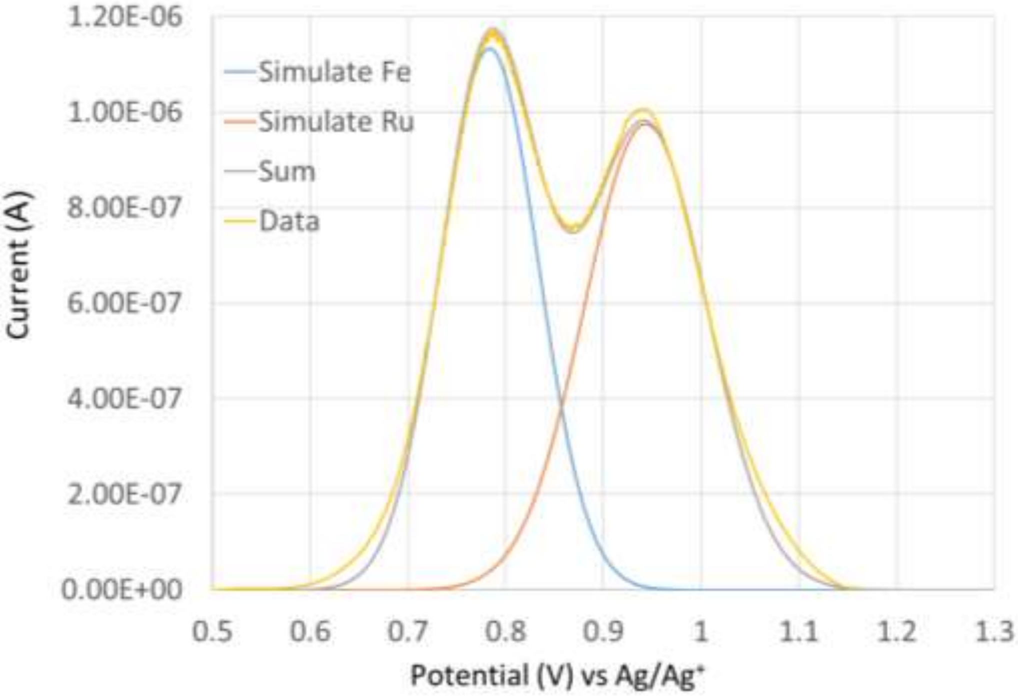

Figure S9. A linear sweep voltammogram of GC/Fe-Ru, **3**, at the scan rate of 1 mVs^{-1} with the simulated signals for the $Fe(tpy)_2$ and $Ru(tpy)_2$ section.

The Γ values of the $Fe(tpy)_2$ and $Ru(tpy)_2$ moieties in the film of **3** are 2.10×10^{-8} mol cm^{-2} and 2.28×10^{-8} mol cm^{-2}, corresponding to 960 and 1040 units, respectively.

Evaluation of the length of 2-Fe and 2-Ru blocks in 4

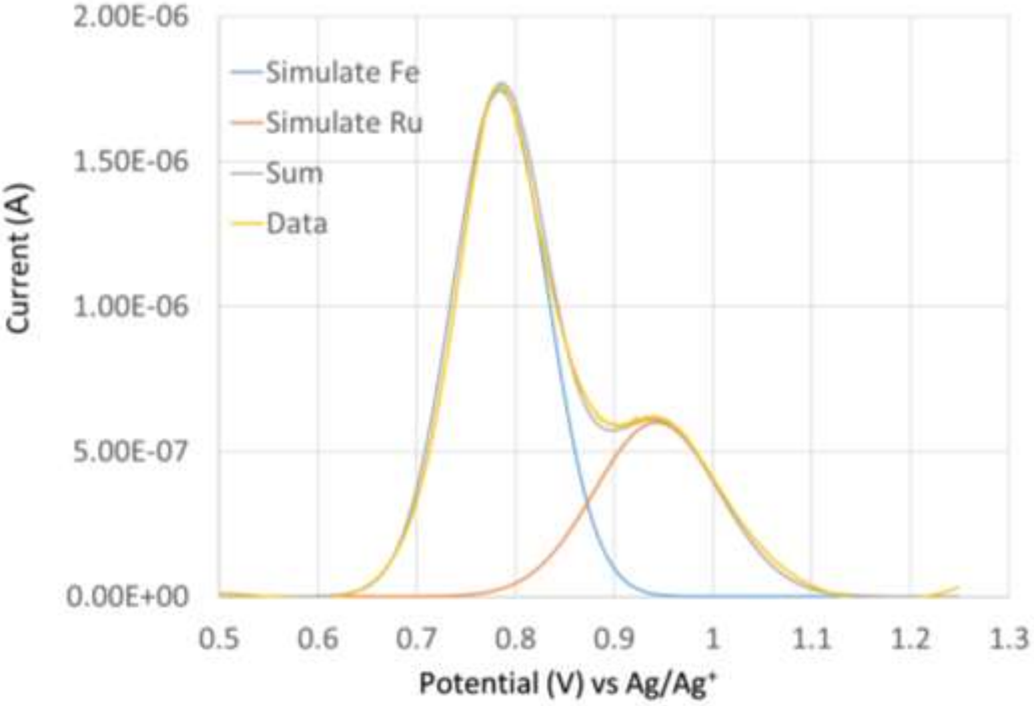

Figure S10. A linear sweep voltammogram of GC/Ru-Fe, **4**, at the scan rate of 1 mVs^{-1} with the simulated signals for the $Fe(tpy)_2$ and $Ru(tpy)_2$ section.

The Γ values of the $Fe(tpy)_2$ and $Ru(tpy)_2$ moieties in the film of **4** are 3.11×10^{-8} mol cm^{-2} and 1.41×10^{-8} mol cm^{-2}, corresponding to1410 and 640 units, respectively.
